# Pifithrin-μ, an Inhibitor of Heat-Shock Protein 70, Can Increase the Antitumor Effects of Hyperthermia Against Human Prostate Cancer Cells

**DOI:** 10.1371/journal.pone.0078772

**Published:** 2013-11-14

**Authors:** Kazumasa Sekihara, Nanae Harashima, Miki Tongu, Yukihisa Tamaki, Nobue Uchida, Taisuke Inomata, Mamoru Harada

**Affiliations:** 1 Department of Immunology, Shimane University Faculty of Medicine, Shimane, Japan; 2 Department of Radiation Oncology, Shimane University Faculty of Medicine, Shimane, Japan; 3 Department of Experimental Animals, Center for Integrated Research in Science, Shimane University, Izumo, Shimane, Japan; 4 Department of Radiation Oncology, Tottori Prefectural Central Hospital, Tottori, Japan; Queensland University of Technology, Australia

## Abstract

Hyperthermia (HT) improves the efficacy of anti-cancer radiotherapy and chemotherapy. However, HT also inevitably evokes stress responses and increases the expression of heat-shock proteins (HSPs) in cancer cells. Among the HSPs, HSP70 is known as a pro-survival protein. In this study, we investigated the sensitizing effect of pifithrin (PFT)-μ, a small molecule inhibitor of HSP70, when three human prostate cancer cell lines (LNCaP, PC-3, and DU-145) were treated with HT (43°C for 2 h). All cell lines constitutively expressed HSP70, and HT further increased its expression in LNCaP and DU-145. Knockdown of HSP70 with RNA interference decreased the viability and colony-forming ability of cancer cells. PFT-μ decreased the viabilities of all cell lines at one-tenth the dose of Quercetin, a well-known HSP inhibitor. The combination therapy with suboptimal doses of PFT-μ and HT decreased the viability of cancer cells most effectively when PFT-μ was added immediately before HT, and this combination effect was abolished by pre-knockdown of HSP70, suggesting that the effect was mediated via HSP70 inhibition. The combination therapy induced cell death, partially caspase-dependent, and decreased proliferating cancer cells, with decreased expression of c-Myc and cyclin D1 and increased expression of p21^WAF1/Cip^, indicating arrest of cell growth. Additionally, the combination therapy significantly decreased the colony-forming ability of cancer cells compared to therapy with either alone. Furthermore, in a xenograft mouse model, the combination therapy significantly inhibited PC-3 tumor growth. These findings suggest that PFT-μ can effectively enhance HT-induced antitumor effects via HSP70 inhibition by inducing cell death and arrest of cell growth, and that PFT-μ is a promising agent for use in combination with HT to treat prostate cancer.

## Introduction

Prostate cancer is the most common cancer and the third most common cause of cancer-related mortality in men in the United States [1]. Although early-stage prostate cancer can be well controlled by surgery or radiotherapy, patients with advanced prostate cancer are treated with hormone therapy [2]. However, after a short-term remission, surviving cancer cells often return with increased malignancy [3]. Therefore, to improve survival in men with prostate cancer, new therapeutic strategies must be developed.

Hyperthermia (HT) is an effective therapy that has low toxicity, mild side-effects, and has been shown to be synergistic with other types of anti-cancer therapies. Numerous *in vitro* and *in vivo* studies have revealed that HT effectively improves the efficacy of radiotherapy and chemotherapy against various types of cancers [4]–[6]. Additionally, many clinical trials have shown that adding HT to radiotherapy or chemotherapy can yield a more complete response [7]–[11]. However, HT is inevitably associated with heat-shock proteins (HSPs) [12], [13]. HSPs are molecular chaperones that act as the primary cellular defense against damage to the proteome, initiating refolding of denatured proteins and regulating degradation after severe protein damage [14]. HSPs protect cells both by limiting the effects of protein-damaging agents through protein chaperoning and refolding and by directly blocking cell death pathways [15]–[18]. Among the HSPs, HSP70 is a stress-inducible HSP that has been reported to play a role in therapy-resistance [19]. In contrast to its very low level in unstressed normal cells, HSP70 expression increases rapidly in response to various stresses [20], [21]. Importantly, increased expression of HSP70 in cancer cells has been reported to be associated with malignant features and poorer prognosis of cancer patients [22]. This evidence suggests that HSP70 is a promising target in cancer treatment. Reducing HSP70 levels in some cultured tumor cells has been reported to induce cell death, and/or to sensitize them to cytotoxic agents, while having no obvious deleterious effects on non-tumor cells [23]–[28].

Pifithrin (PFT)-μ (2-phenylethynesulfonamide) was initially identified as a small-molecule inhibitor of binding of p53 to mitochondria [29]. Thereafter, this molecule was found to selectively interact with HSP70 and to inhibit its functions [30]. This information led us to test the hypothesis that PFT-μ could enhance HT-induced antitumor effects against human prostate cancer cells. In the current study, after confirming that HSP70 is constitutively expressed and/or enhanced by HT and plays a pro-survival role in human prostate cancer cells, we demonstrated that the combination of suboptimal doses of PFT-μ can efficiently enhance HT-induced antitumor effects against human prostate cancer *in vitro*, and that the combination therapy can inhibit tumor growth in a xenograft mouse model.

## Materials and Methods

### Cell culture and reagents

Three human prostate cancer cell lines (LNCaP, PC-3, and DU-145) were obtained from the American Type Culture Collection (ATCC, Rockville, MD, USA), and were maintained in RPMI 1640 medium (Sigma-Aldrich, St. Louis, MO, USA) supplemented with 10% fetal bovine serum (Invitrogen, Grand Island, NY, USA) and 20 µg/ml gentamicin (Sigma-Aldrich) at 37°C in a humidified atmosphere containing 5% CO_2_. PFT-μ and Quercetin were purchased from Santa Cruz Biotechnology (SCB: Santa Cruz, CA, USA) and Cayman Chemical Company (Ann Arbor, MI, USA), respectively.

### Cell viability assay

Cell viability was evaluated using the 2-(2-methoxy-4-nitrophenyl)-3-(4-nitrophenyl)-5-(2, 4-disulfophenyl)-2H-tetrazolium monosodium salt (WST-8) assay (Nacalai Tesque, Kyoto, Japan). Briefly, cells were seeded in flat-bottomed 96-well plates. Two days later, WST-8 was added to each well, and the plates were read at a wavelength of 450 nm after 3 h.

### Combination therapy with HT and PFT-μ

To induce HT, cancer cells, which were seeded 1 day before, were incubated at 43°C for 2 h. Cancer cells were always cultured in the presence of PFT-μ for 2 days. In some experiments, HT was performed on day 1, 2, or 3 after starting culture of cancer cells.

### Transfection of small interfering RNA (siRNA)

Transfection of siRNA was performed using Lipofectamine™ RNAiMAX (Invitrogen), according to the manufacturer's instructions. HSP70 siRNA (sc-29352) was purchased from SCB. Control siRNA (#6568) was purchased from Cell Signaling Technology (CST), Danvers, MA, USA. Three days after siRNA transfection, the cancer cells were used for the experiments.

### Colony-forming assay

Cells were seeded in flat-bottomed six-well plates. One day later, PFT-μ was added and treated with or without HT. Two days after the addition of PFT-μ, the medium was replaced with new medium containing no PFT-μ, and the culture was continued for an additional 10–12 days. Thereafter, colonies were fixated with methanol and stained with 0.05% crystal violet, then counted.

### Immunoblot

Cells were lysed with a mammalian protein extraction reagent (M-PER; Thermo Scientific, Rockford, IL, USA) containing a protease inhibitor cocktail (Nacalai Tesque). Equal amounts of protein were resolved on 4–12% gradient or 12% SDS-PAGE gels, and then transferred to polyvinylidene fluoride membranes. After blocking the membranes, the blots were incubated with the following primary antibodies: anti-HSP70 (HSP72; R&D systems, Minneapolis, MN, USA), anti-HSP90 (CST), anti-c-Myc (Epotomics, Burlingame, CA, USA), anti-cyclinD1 (CST), anti-β-actin (BioLegend, San Diego, CA, USA) and anti-α-tubulin (SCB). Goat anti-rabbit or goat anti-mouse alkaline phosphatase-conjugated secondary antibodies (Invitrogen) were used to detect the primary antibodies. To evaluate the expression level of HSP70 after HT, a ratio of the expression of HSP70/the expression of β-actin was evaluated by densitometry using the ImageJ (http://rsbwed.nih.gov/ij/).

### Flow cytometric analysis

Cell death was assessed by using the Annexin V-FITC Apoptosis Detection Kit (BioVision, Mountain View, CA, USA), APC-conjugated Annexin V (BD Biosciences, San Jose, CA, USA), and propidium iodide (PI). For inhibition of caspases, z-VAD-fmk (R&D Systems, Mineapolis, MN, USA) was added, and DMSO was used as a vehicle control. To examine the cell cycle and proliferation of cancer cells, a BrdU/7AAD Proliferation Kit (Becton Dickinson, Fullerton, CA, USA) was used according to the manufacturer's instructions. Analysis was performed using a FACSCalibur flow cytometer (Becton Dickinson).

### *In vivo* xenograft model

BALB *nu/nu* male mice, purchased from CLEA Japan (Tokyo, Japan), were maintained under specific-pathogen-free conditions. The protocol was approved by the Committee on the Ethics of Animal Experiments of the Shimane University Faculty of Medicine (Permit Number: IZ25-6). All efforts were made to minimize suffering. Mice were inoculated in the right footpad with 1×10^6^ PC-3 cells with Matrigel (Japan BD Biosciences, Tokyo, Japan) at a 1∶1 volume ratio in a volume of 50 µl. On day 15, the mice were pooled and divided into four groups. On days 0 and 4 after grouping, these PC-3-bearing mice were treated with PFT-μ and/or HT. PFT-μ (100 µg in 50 µl) was injected into the tumor. As a vehicle control, 50 µl of DMSO were injected. To perform HT therapy, these PC-3-bearing mice had only their footpads bathed in 43°C water for 30 min after fixing them on plates with tape. Subsequently, the tumor size, the product of two perpendicular diameters, and the footpad thickness were measured twice weekly.

### Statistical analyses

Data were evaluated statistically using an unpaired two-tailed Student's *t*-test or a parametric Dunnett test. A *P* value of less than 0.05 was considered to indicate statistical significance.

## Results

### Pro-survival role of HSP70 in human prostate cancer cells

**Figure 1:**
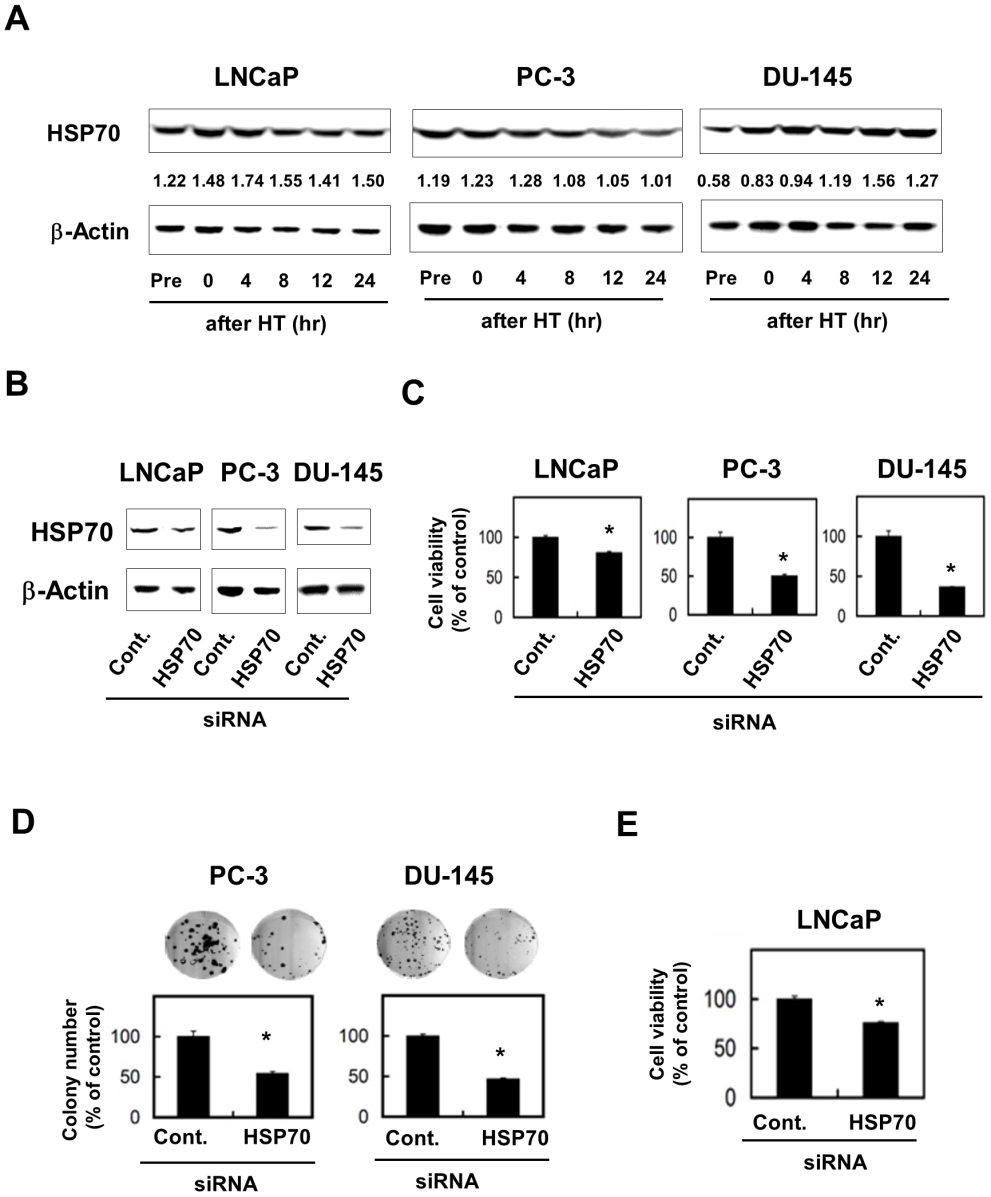
Pro-survival role of HSP70 in human prostate cancer cell lines. (**A**) Three prostate cancer cell lines were treated with HT (43°C for 2 h). Before and after 4, 8, 12, and 24 h, lysates were prepared and the expression of HSP70 was assessed by immunoblot. β-Actin was used as the control. The number represents the ratio of the expression of HSP70 to that of β-actin, which was evaluated by densitometry using ImageJ. (**B**) Three days after transfection of HSP70 siRNA or control siRNA, the expression of HSP70 was assessed by immunoblot. β-Actin was used as a control. (**C**) Three cell lines, which had been pre-transfected with HSP70 siRNA or control siRNA three days before, were cultured. After 48 h, cell viability (%) was determined using the WST-8 assay. The results are shown as the mean ± SD of three wells. * *P*<0.05 (Student's *t*-test) (**D**) PC-3 and DU-145, which had been pre-transfected with HSP70 siRNA or control siRNA 2 days prior, were cultured for the colony-formation assay for 12 days. The results are shown as the mean ± SD of three wells. * *P*<0.05 (Student's *t*-test) (**E**) LNCaP, which had been pre-transfected with HSP70 siRNA or control siRNA 2 days prior, were cultured for 12 days, and cell viability (%) was determined using the WST-8 assay. The results are shown as the mean ± SD of three wells. * *P*<0.05 (Student's *t*-test).

We first assessed the HSP70 protein levels in three human prostate cancer cell lines before and after treatment with HT (43°C for 2 h) (Fig. 1A). These cell lines constitutively expressed HSP70. Although no definite change was observed in PC-3, the expression levels in LNCaP and DU-145 were increased after HT. We next determined whether HSP70 plays a pro-survival role in prostate cancer cells. The expression of HSP70 was selectively decreased by transfection of HSP70 siRNA (Fig. 1B), which decreased the cell viability of all three cell lines (Fig. 1C). We also found that knockdown of HSP70 significantly decreased the colony-forming ability of PC-3 and DU-145 (Fig. 1D). Additionally, because of the inability of LNCaP to form colonies in this assay (data not shown), their viability after a 12-day culture was evaluated. As a result, knockdown of HSP70 decreased the viability of LNCaP cells after the 12-day culture (Fig. 1E). These results indicate that HSP70 plays a pro-survival role in human prostate cancer cells.

### Antitumor effects on human prostate cancer cells induced by the combination therapy with HT and PFT-μ

**Figure 2:**
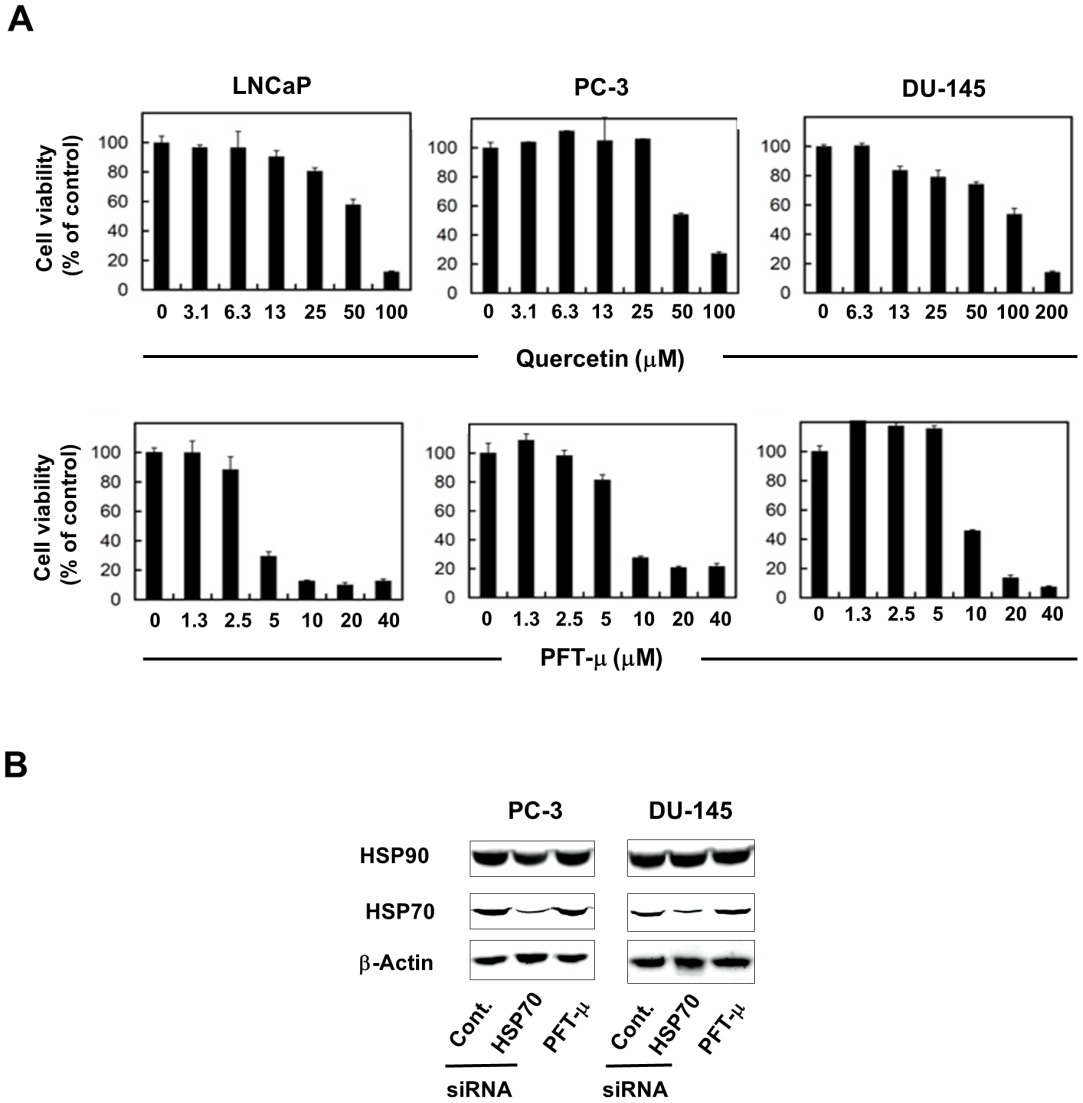
Effects of Quercetin and PFT-μ on the viability of prostate cancer cells. (**A**) Three cell lines were cultured with the indicated doses of Quercetin or PFT-μ. After 48 h, cell viability (%) was determined using the WST-8 assay. The results are shown as the mean ± SD of three wells. (**B**) Two days after transfection of HSP70 siRNA or control siRNA, the expression of HSP90 and HSP70 was assessed by immunoblot. The expression of these HSPs was also examined after the treatment with PFT-μ (5 µM). β-Actin was used as a control.

The above data suggest that inhibition of HSP70 could facilitate treatment of prostate cancer using HT. Therefore, we next investigated the effect of the new HSP70 inhibitor PFT-μ on HT-induced antitumor activity. Before evaluating the antitumor effect induced by the combination of HT and PFT-μ, we compared the antitumor effect of PFT-μ to that of Quercetin, a heat-shock factor (HSF)-1 inhibitor, which inhibits the up-regulation of all heat-shock-induced genes, including *HSP70* gene [31] (Fig. 2A). Both Quercetin and PFT-μ decreased the viability of three prostate cancer cell lines in a dose-dependent manner, but PFT-μ exerted its antitumor effect at almost one-tenth the dose of Quercetin. We also confirmed that knockdown of HSP70 failed to influence the expression of HSP90, another key HSP of the stress response pathway (Fig. 2B). PFT-μ had no effect on the HSP70 protein expression, as reported previously [30].

**Figure 3:**
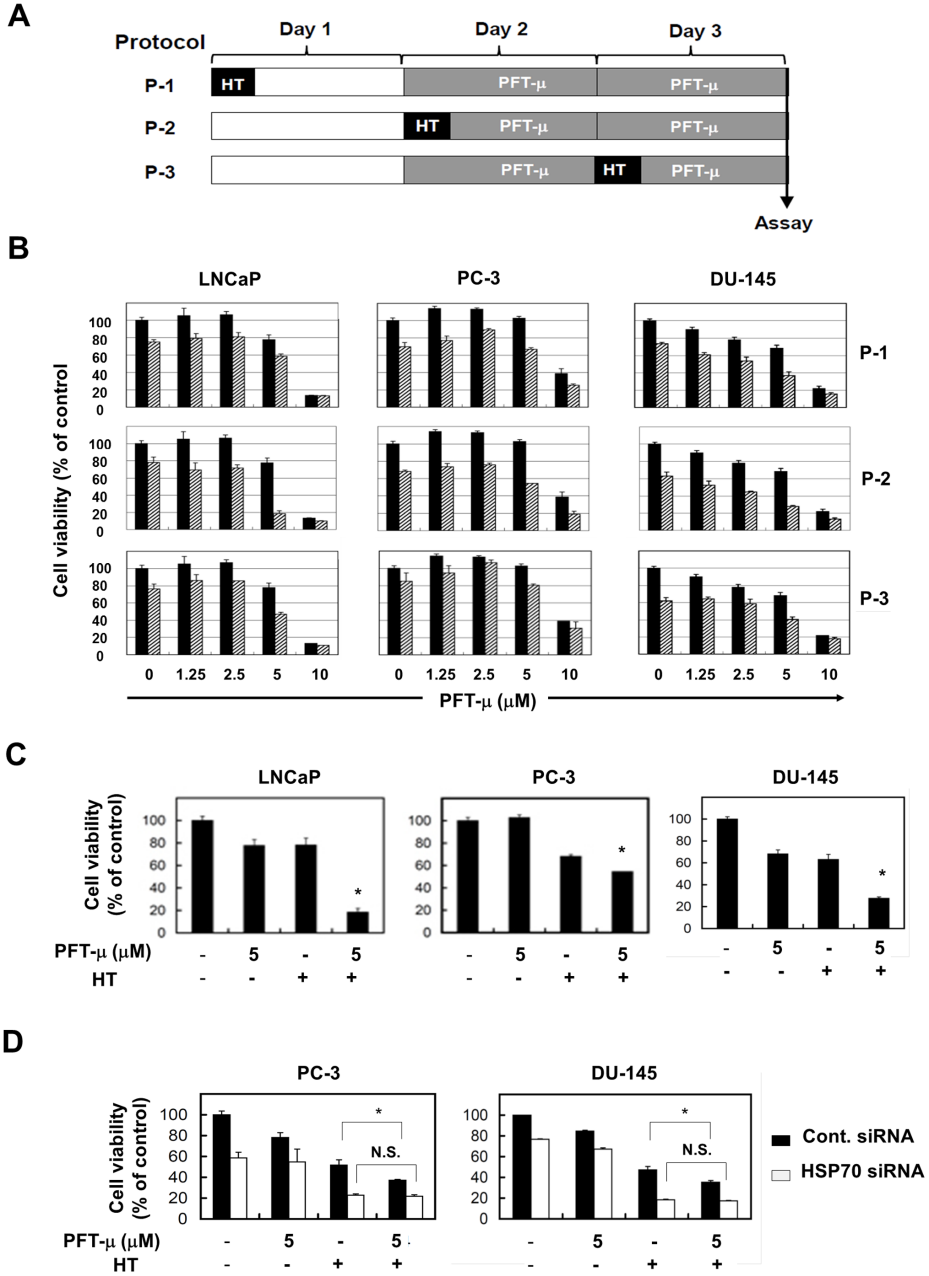
Effects of the combination of HT and PFT-μ on prostate cancer cells. (**A**) Three different protocols are shown. (**B**) Three cell lines were cultured with the indicated doses of PFT-μ based on three different protocols with HT (striped bar) or without HT (black bar). HT was performed at 43°C for 2 h. After 48 h, cell viability (%) was determined using the WST-8 assay. The results are shown as the mean ± SD of three wells. (**C**) Selected results are shown. The results are shown as the mean ± SD of three wells. * *P*<0.05 (Student's *t*-test) (**D**) PC-3 and DU-145, which had been pre-transfected with HSP70 siRNA or control siRNA 2 days earlier, were culured with PFT-μ (5 µM) with/without HT (43°C for 2 h). After 48 h, cell viability (%) was determined using the WST-8 assay. The results are shown as the mean ± SD of three wells. * *P*<0.05 (Student's *t*-test) N.S., not significant.

Then, we assessed the antitumor effects induced by the combination of HT and PFT-μ. We first explored for the optimal protocols to maximize the antitumor effects induced by this combination. As shown in Fig. 3A, three human prostate cancer cell lines were treated with three different protocols; in protocol-1 (P-1), HT was performed 24 h before the addition of PFT-μ; in protocol-2 (P-2), PFT-μ was added immediately before HT; and in protocol-3 (P-3), HT was performed 24 h after the addition of PFT-μ. The most prominent effect was observed upon application of the P-2 protocol (Fig. 3B). Selected representative data are shown in Fig. 3C. The viability of cancer cells was decreased significantly when HT was combined with a suboptimal dose (5 µM) of PFT-μ. We further determined whether the combination effect could be observed in cancer cells that were pre-transfected with HSP70 siRNA (Fig. 3D); the pre-knockdown of HSP70 abolished the combination effect against PC-3 and DU-145. These results indicate that suboptimal doses of PFT-μ can enhance HT-induced antitumor effects on prostate cancer cells via HSP70 inhibition, and that the most prominent effect is achieved when PFT-μ is added immediately before HT.

### Cancer cell death after the combination therapy of HT and PFT-μ

**Figure 4:**
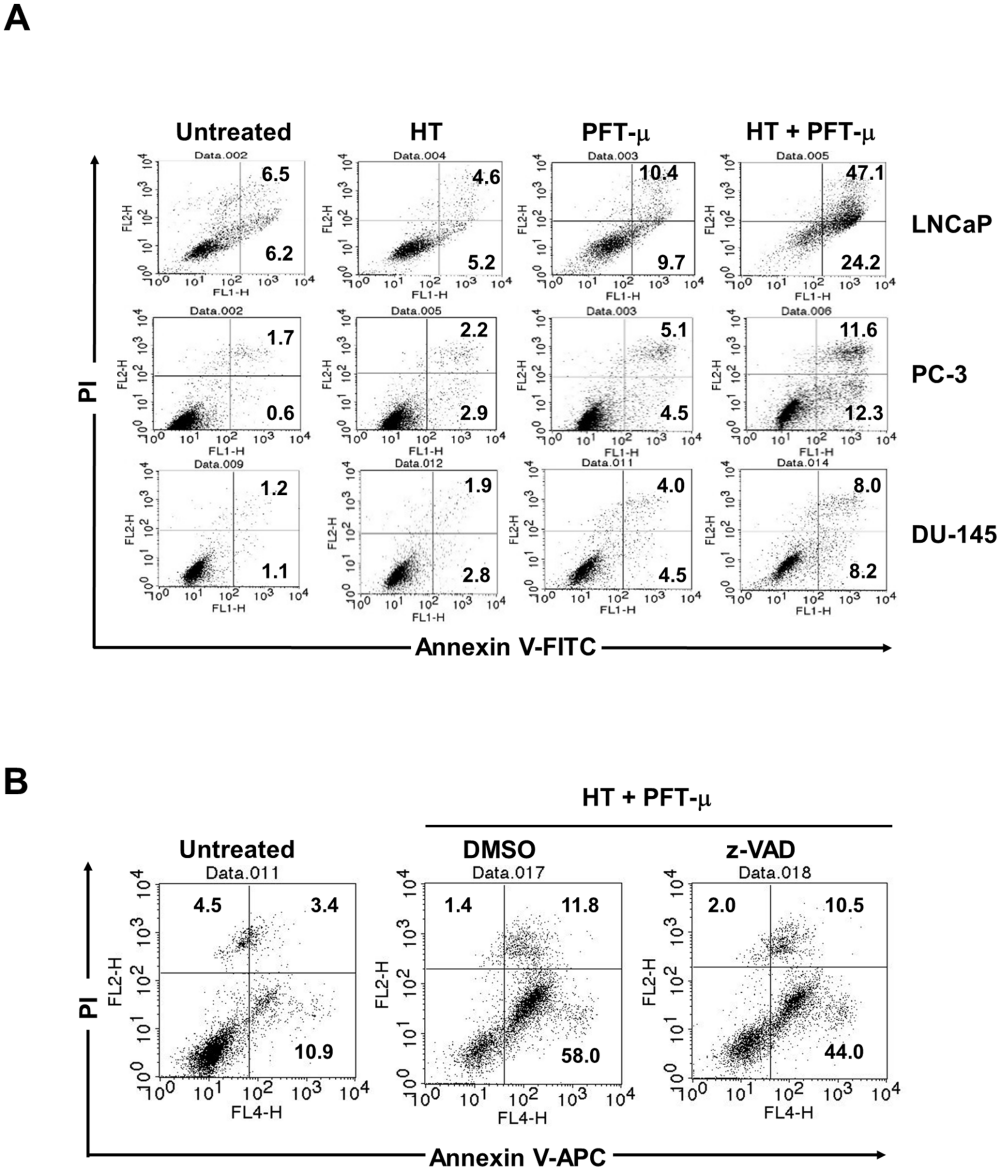
Cancer cell death after combination therapy with HT and PFT-μ. (**A**) Three cell lines were treated with HT (43°C for 2 h) and/or PFT-μ. After 48 h, flow cytometry was performed after staining with FITC-conjugated Annexin V and PI. A representative result is shown. The numbers represent the percentages of each subset. (**B**) LNCaP cells were treated with both HT and PFT-μ (5 µM) in the presence of z-VAD (20 µM) or control DMSO. After 48 h, flow cytometry was performed after staining with APC-conjugated Annexin V and PI. The numbers represent the percentages of each subset.

In the studies described above, we evaluated mainly the antitumor effects on prostate cancer cells by measuring viability 2 days after treatment with HT and PFT-μ. However, such effects on viability may reflect alterations in cell death and/or growth. Therefore, we next assessed the underlying mechanism of action. As shown in Fig. 4A, HT alone failed to increase the percentage of Annexin-V^+^ cells, whereas treatment with PFT-μ increased it slightly. However, the combination treatment drastically increased the percentage of Annexin V^+^ cells, both early apoptotic Annexin-V^+^/PI^−^ and late apoptotic and/or necrotic Annexin-V^+^/PI^+^, especially in LNCaP cells. The increase in the percentage of Annexin V^+^ cells seemed to be only additive in PC-3 and DU-145. Additionally, adding 20 µM z-VAD, a pan-caspase inhibitor, partially reduced the percentage of Annexin V^+^ cells in LNCaP after combination treatment of HT and PFT-μ (Fig. 4B). The z-VAD-induced recovery from Annexin V^+^ cells was detected in the Annexin V^+^/PI^−^ (early apoptotic) subset. This dose of z-VAD was sufficient to inhibit TRAIL-induced cell death of death receptor-expressing human pancreatic cancer cells [32]. These results indicate that, although the efficacy varies among cancer cell lines, combination therapy with HT and PFT-μ can enhance death of prostate cancer cells, and that the combination therapy-induced cell death of LNCaP is partially caspase-dependent.

### Combination therapy with HT and PFT-μ can arrest the growth of prostate cancer cells

**Figure 5:**
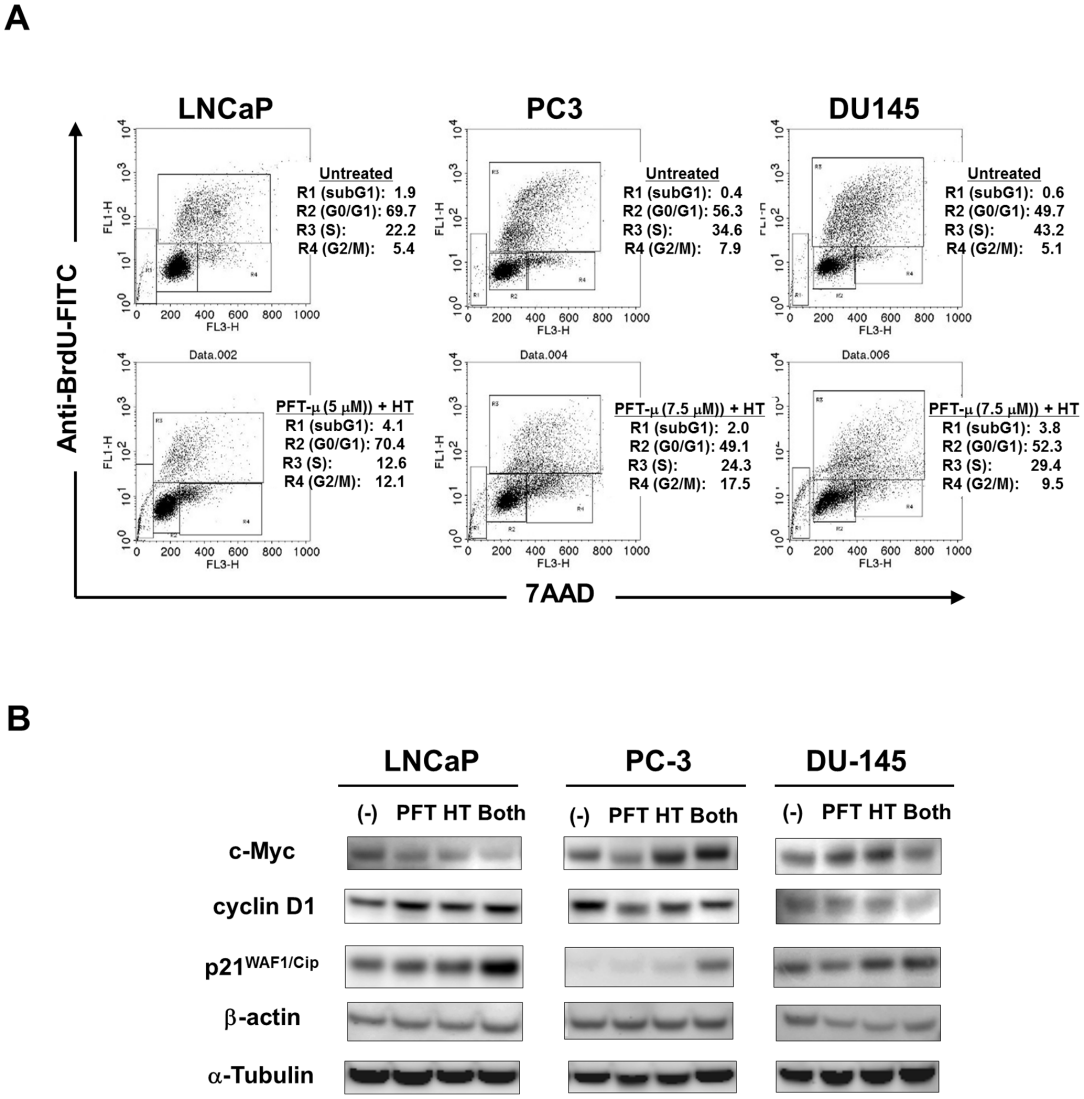
Combination therapy with HT and PFT-μ arrested the growth of prostate cancer cells. (**A**) Three cell lines were treated with or without HT (43°C for 2 h) and PFT-μ (5 µM) for 2 days. During the last 5 h for LNCaP, 90 min for PC-3, and 3 h for DU-145, cells were cultured with BrdU (10 µM). Then, harvested cells were stained with FITC-conjugated anti-BrdU antibody and 7AAD, and flow cytometry was performed. Numbers represent the percentages of each subset. (**B**) Three cell lines were treated with either or both of HT and PFT-μ (5 µM) for 2 days, and the expression levels of c-Myc, cyclin D1, and p21^WAF1/Cip^ protein were determined by immunoblot. β-Actin and α-tubulin were used as controls.

We further investigated whether cell growth arrest was involved in the antitumor effects induced by combination therapy with HT and PFT-μ. As shown in Fig. 5A, we assessed the proliferation and cell cycle of cancer cells by evaluating BrdU uptake and 7AAD staining. We found that combination therapy with HT and PFT-μ decreased the percentage of BrdU^+^ S-phase cancer cells and increased that of G2/M phase cancer cells in the three cell lines. We also assessed the expression of cell cycle-related molecules and found that the combination therapy resulted in decreased expression of c-Myc in LNCaP and decreased expression of cyclin D1 in PC-3 and DU-145. Additionally, the expression of p21^WAF1/Cip^ was increased in the three cancer cell lines. These data suggest that cell growth arrest contributes to the antitumor effects induced by combination therapy with HT and PFT-μ.

### Combination therapy with HT and PFT-μ can decrease the colony-forming ability of cancer cells

**Figure 6:**
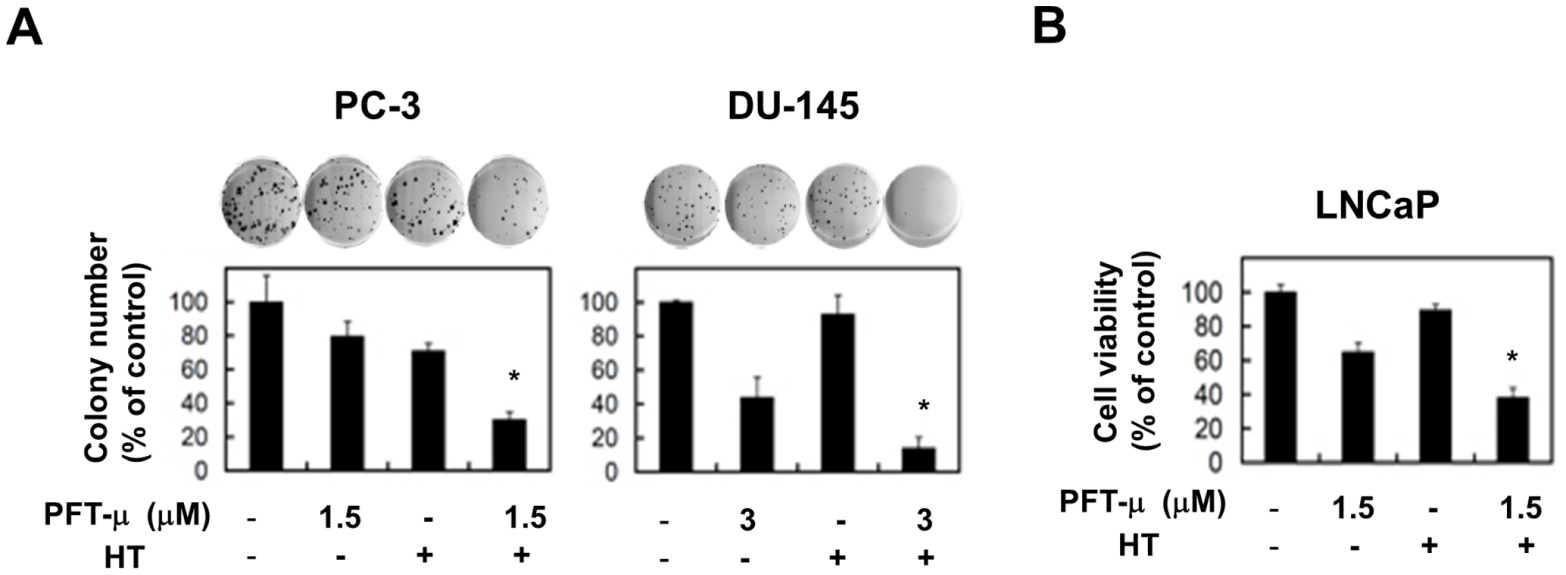
Combination therapy with HT and PFT-μ decreased the colony-forming ability of cancer cells. (**A**) PC-3 and DU-145 cells were cultured for 14 days and their colony-formation capacities were determined. The results are shown as the mean ± SD of three wells. * *P*<0.05 (Student's *t*-test) (**B**) LNCaP cells were cultured for 12 days, and viability (%) was determined using the WST-8 assay. The results are shown as the mean ± SD of three wells. * *P*<0.05 (Student's *t*-test).

We next investigated the effect of combination therapy with HT and PFT-μ on the colony-forming ability of prostate cancer cells. The combination therapy significantly decreased the colony-forming ability of PC-3 and DU-145 cells (Fig. 6A) and decreased the viability of LNCaP cells in the long-term (12-day) culture (Fig. 6B). These results indicate that combination therapy with HT and PFT-μ can decrease the colony-forming ability and viability, in a long-term culture, of prostate cancer cells.

### *In vivo* antitumor effect of HT and PFT-μ combination therapy in a xenograft mouse model

**Figure 7:**
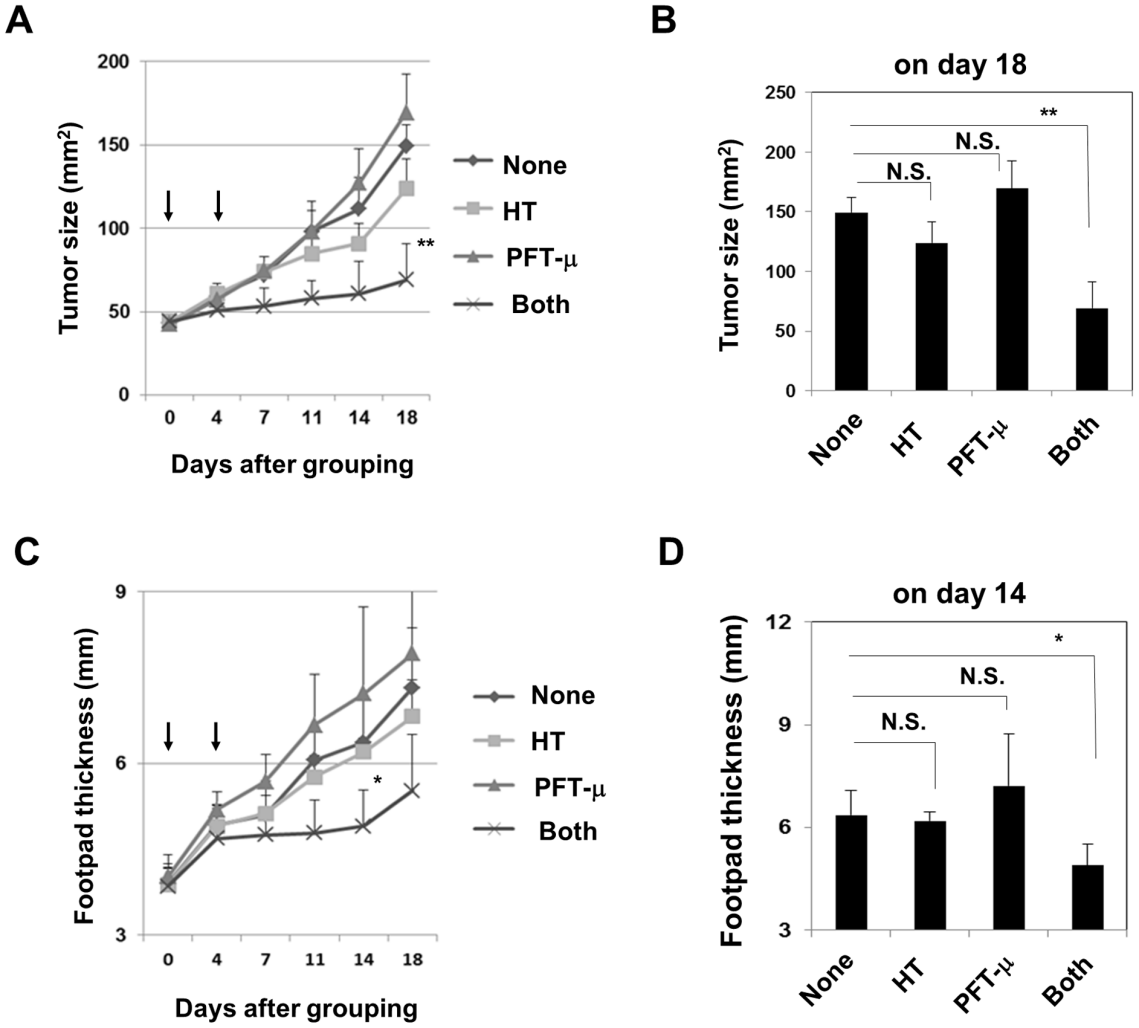
*In vivo* antitumor effects of PFT-μ and HT in a xenograft mouse model. BALB *nu/nu* male mice were inoculated in the right footpad with 1×10^6^ PC-3 cells with Matrigel. On day 15, the mice were pooled and divided into four groups. Each group contained six mice. On days 0 and 4 after grouping, the PC-3-bearing mice were locally injected with PFT-μ (100 µg in 50 µl) and/or treated with HT (43°C for 30 min). Arrows represent the day of treatment. As a vehicle control, 50 µl of DMSO were injected. HT was performed 1 h after the local injection of PFT-μ. Thereafter, the tumor size, the product of two perpendicular diameters (**A, B**), and the footpad thickness (**C, D**) were measured twice weekly. The results are shown as the mean ± SD of six mice. *P<0.05 ** *P*<0.01 (Dunnett test) N.S., not significant.

Finally, we evaluated whether combination therapy with HT and PFT-μ exerted an antitumor effect on established human prostate cancer in a xenograft mouse model (Fig. 7). The footpads of nude mice were inoculated with PC-3 cells and the mice were treated with PFT-μ and/or HT. Given the anatomy of the footpad, the tumor growth was evaluated by measuring not only the product of two perpendicular diameters but also the footpad thickness. As a result, although the local administration of PFT-μ had no antitumor effect but, rather, promoted the tumor growth, and HT decreased the tumor growth slightly but not significantly, the combination therapy with HT and PFT-μ significantly suppressed the tumor growth compared with the control group.

## Discussion

Among a variety of cancer types, HT is applicable especially to prostate cancer [33]. However, as discussed in the Introduction, HT inevitably evokes stress responses in cancer cells. HSP70 is a potent heat-inducible pro-survival protein that confers cytoprotection against various death-inducing stimuli and increases tumorigenicity [25], [27], [34]. Therefore, HSP70 has been suggested as a promising target for cancer treatment [19]. In the current study, we demonstrated that three prostate cancer cell lines are constitutively positive for HSP70, and that HT can increase its expression in LNCaP and DU-145 cell lines. Unexpectedly, the expression of HSP70 in PC-3 was slightly decreased 8 h after HT; we are unable to explain this observation at present. However, it is of note that selective knockdown of HSP70 in cancer cells significantly decreased their viability and colony-forming ability. Our results suggest that HSP70 is a pro-survival protein in prostate cancer cells, consistent with previous reports [23]–[28]. These findings led us to test the effect of the combination of HT with PFT-μ, a recently identified HSP70 inhibitor [30], on human prostate cancer cells.

Combination therapy with HT and PFT-μ significantly decreased the viability of three prostate cancer cell lines compared to treatment with either therapy alone. Regarding the underlying mechanism, we tested two possibilities; *i.e.*, cell death and cell growth arrest. Annexin V/PI staining revealed that the combination therapy increased the percentage of Annexin V^+^ cells (Fig. 4A). Although the combination effect was slight in PC-3 and DU-145 cells, a drastic and synergistic increase in the percentage of Annexin V^+^ cells was observed in LNCaP cells. HT alone did not affect the percentage of Annexin V^+^ cells, and the effect of PFT-μ alone was also marginal. Annexin V^+^/PI^+^ positivity does not indicate apoptotic cells because late-necrotic cells are also positive for both Annexin V and PI. With regard to cell growth arrest, the combination therapy decreased the S-phase fraction and increased the G2/M-phase fraction in three cancer cell lines (Fig. 5A). These results are consistent with those from a recent report showing that PFT-μ can induce G2/M arrest in cancer cells [35]. Additionally, the combination therapy decreased the expression of c-Myc in LNCaP and cyclin D1 in PC-3 and DU-145, and increased the expression of p21^WAF1/Cip^ in three cell lines. These changes in cell cycle-related molecules may partially account for the arrest of the growth of prostate cancer cells in response to the combination therapy.

HSP90 and HSP70 influence each other [36]. Since HSP90 inhibition is associated with the up-regulation of HSP70 [37], multiple groups have reported that co-inhibition of HSP70 markedly enhances the cytotoxicity of HSP90 inhibitors for several different tumor types [38]. Conversely, HSP70 is a critical co-chaperon for HSP90 and is involved in the delivery of client proteins to HSP90 [39], and HSP70 inhibition can induce tumor-specific apoptosis via HSP90 function [28]. These lines of information suggest that knockdown of HSP70 and PFT-μ enhanced the effect of HT via the inhibition of HSP90. Therefore, we examined the effect of knockdown of HSP70 or PFT-μ on the expression of HSP90 in cancer cells, while no change in the HSP90 expression was observed (Fig. 2B). However, this result cannot exclude the possibility that inhibition of HSP70 or PFT-μ influenced the sequestration of HSP90 client proteins, including epidermal growth factor receptor, HER2/ErbB2 and AKT, into an insoluble fraction and promoted their aggregation and inactivation, as reported [40].

We determined whether the combination therapy-induced cell death of LNCaP cells was dependent on caspase (Fig. 4B). The addition of the pan-caspase inhibitor z-VAD decreased the percentage of Annexin V^+^/PI^−^ early apoptotic LNCaP cells, implying that caspase-dependent apoptosis was partially responsible for the combination therapy-induced cell death of LNCaP. Given that HT alone failed to induce cell death, this effect seems to reflect mainly the effect of PFT-μ. We reported recently that PFT-μ causes caspase-dependent and -independent cell death of human pancreatic cancer cells [32]. Regarding caspase-independent cell death, HSP70 has been reported to bind to apoptosis-inducing factor (AIF), which induces caspase-independent apoptosis by translocation into the nucleus [41]. However, RNA interference of AIF had no effect on PFT-μ–induced cell death of prostate cancer cells (data not shown). Thus, AIF likely does not participate in the combination therapy-induced death of LNCaP cells. Alternatively, HSP70 has been reported to localize to the membranes of lysosomes, promote cancer cell viability, and inhibit TNF-induced cell death by inhibiting lysosomal membrane permeability [42]. Thus, HSP70 enhances survival by stabilizing the lysosomes in cancer cells. Since PFT-μ binds HSP70, it is possible that PFT-μ inhibits HSP70-induced stabilization of lysosomal membrane permeability, resulting in increased cell death. However, further study is needed to elucidate the precise mechanism of cell death after combination therapy with HT and PFT-μ.

When HT is combined with anti-cancer drugs, the timing of administration of the drug is critical [13]. Therefore, in this study, we compared the antitumor effects induced by three different protocols, and found that that HT immediately after the addition of PFT-μ yielded the greatest antitumor effects (Fig. 3). This indirectly suggests that HSP70 plays a protective role immediately after heat stress.

We evaluated the antitumor effects of the combination therapy by colony-formation assay. This assay was performed for 12 days, and the result may reflect the effect of both cell death and cell growth arrest. The combination with HT and PFT-μ significantly decreased the colony-forming ability of PC-3 and DU-145 cells. In the case of LNCaP, the combination therapy caused an antitumor effect on LNCaP in a long-term (12-day) cell viability assay. In these assays, PFT-μ was removed after the initial 2-day culture because the continuous presence of PFT-μ, even at the relatively low dose used for the short-term cell viability assay, was too high to allow colonies to form. We assume that the colony-formation assay and the long-term (12-day) cell viability assay are useful for testing the long-lasting effects on cancer cells after transient anti-cancer therapy.

Although Quercetin is a well-known HSP inhibitor [31], this drug has not been used clinically because relatively high doses are needed to elicit antitumor effects *in vivo*. We compared the antitumor effects of Quercetin and PFT-μ and found that PFT-μ can induce the antitumor effect even at one-tenth the dose of Quercetin (Fig. 2A). This suggests that PFT-μ effectively enhances the antitumor effects of HT on human prostate cancer cells.

In conclusion, we investigated the sensitizing effect of PFT-μ, a small molecule inhibitor of HSP70, when human prostate cancer cells were treated with HT. Our findings suggest that PFT-μ effectively enhances HT-induced antitumor effects both *in vitro* and *in vivo*, and that PFT-μ is a promising agent for use in combination with HT to treat prostate cancer.
